# Novel high‐docosahexaenoic‐acid tuna oil supplementation modulates gut microbiota and alleviates obesity in high‐fat diet mice

**DOI:** 10.1002/fsn3.1941

**Published:** 2020-11-07

**Authors:** Jing Zhang, Congmin Yi, Jiaojiao Han, Tinghong Ming, Jun Zhou, Chenyang Lu, Ye Li, Xiurong Su

**Affiliations:** ^1^ State Key Laboratory for Quality and Safety of Argo‐products Ningbo University Ningbo China; ^2^ School of Marine Science Ningbo University Ningbo China; ^3^ Faculty of Food Science Zhejiang Pharmaceutical College Ningbo China

**Keywords:** dietary obesity, gut microbiota, novel high‐DHA tuna oil

## Abstract

Studies have documented the benefits of fish oil in different diseases because of its high n‐3 polyunsaturated fatty acid content. However, these studies mostly used commercially available fish oil supplements with a ratio of 18/12 for eicosapentaenoic acid and docosahexaenoic acid (DHA). However, increasing DHA content for this commonly used ratio might bring out a varied metabolic effect, which have remained unclear. Thus, in this study, a novel tuna oil (TO) was applied to investigate the effect of high‐DHA content on the alteration of the gut microbiota and obesity in high‐fat diet mice. The results suggest that high‐DHA TO (HDTO) supplementation notably ameliorates obesity and related lipid parameters and restores the expression of lipid metabolism‐related genes. The benefits of TOs were derived from their modification of the gut microbiota composition and structure in mice. A high‐fat diet triggered an increased *Firmicutes*/*Bacteroidetes* ratio that was remarkably restored by TOs. The number of obesity‐promoting bacteria—*Desulfovibrio*, *Paraeggerthella*, *Terrisporobacter*, *Millionella*, *Lachnoclostridium*, *Anaerobacterium*, and *Ruminiclostridium—*was dramatically reduced. *Desulfovibrio desulfuricans*, *Alistipes putredinis*, and *Millionella massiliensis*, three dysbiosis‐related species, were especially regulated by HDTO. Regarding the prevention of obesity, HDTO outperforms the normal TO. Intriguingly, HDTO feeding to HFD‐fed mice might alter the arginine and proline metabolism of intestinal microbiota.

## Graphical abstract

The high‐DHA tuna oil (TO) did produce different influence on the mice fed with high‐fat diet. The benefits of TOs were derived from their modification of the gut microbiota composition and structure in mice.

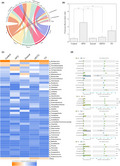

## 1 INTRODUCTION

Obesity has become a worldwide epidemic. According to a report, more than 2.1 billion people have a body mass index ≥25.0 and ≥30.0, and more than half a billion of them with no distinction of age or sex worldwide (Jérôme et al., 2019). This increasing prevalence has become a global health concern and is causing a substantial socioeconomic burden. Obesity, characterized by fat accumulation, chronic inflammation, or impaired satiety signaling, is linked to various chronic diseases, such as cardiovascular diseases, type 2 diabetes, hypertension, and several forms of cancer (Fabbrini et al., 2010; Kahn, 2008; Lavanya & Rana, 2019; Lee et al., 2013). Then, what are the factors resulting in obesity? Host‐microbial interactions have been documented in studies of obesity‐related diseases (Cani et al., 2012). In addition to genetic susceptibility, environmental impact, lack of physical activity and other factors, the greatest risk is the expansion of high‐fat/high‐sugar diets (Crinò et al., 2018; Stoner et al., 2016; Zhang & Yang, 2016). However, the gut microbiota is a key interface for energy acquisition of the host (Ikuo et al., 2013; Mayu et al., 2015). Accumulating evidence has indicated that high‐fat‐diet‐triggered obesity leads to alterations in the gut microbial composition, as well as reductions in microbial diversity and changes in specific bacterial taxa (Daniel et al., 2014; Turnbaugh et al., 2006, 2009; Zhu et al., 2018). These variations in the gut microbiota might result in gut microbial dysbiosis and play an important role in the pathogenesis of obesity. Thus, developing a new strategy to treat obesity surrounding manipulations of the gut microbiota and its metabolites has become a primary public health goal.

Diet may reshape the gut microbiota (Sharma & Tripathi, 2019; Yang & Yu, 2018). With this opinion established, more studies have focused on food‐derived bioactive compounds to provide a new strategy for obesity intervention. Fish oil has long been utilized as a functional food to improve insulin sensitivity and has potent anti‐inflammatory, hypolipidemic, cardiovascular protection, and body weight‐reducing effects due to the presence of n‐3 polyunsaturated fatty acids (n‐3 PUFAs), mainly eicosapentaenoic acid (EPA, 20:5 n‐3) and docosahexaenoic acid (DHA, 22:6 n‐3; da Cunha de Sá et al., 2016; Flachs et al., 2009; Rokling‐Andersen et al., 2009; Saraswathi et al., 2007; Serhan, 2014; Sundaram et al., 2015). However, most of the currently available studies investigating the effect of dietary n‐3 PUFAs employed commercially available fish oil supplements, which are characterized by higher amounts of EPA than DHA, in a distinctive ratio of 18/12 (Fard et al., 2019; Turchini et al., 2009). Nonetheless, the molecular structures of DHA and EPA are different. DHA contains a longer carbon chain (22 vs. 20) and an additional double bond (6 vs. 5) per molecule compared with EPA, which might result in the different metabolic effects between the two molecules. Moreover, increasing evidence has shown that EPA and DHA exert heterogeneous effects on human health; dietary DHA or EPA have different metabolic fates in animal models; for example, DHA is preferentially retained over EPA, and EPA is β‐oxidized more than DHA (Ghasemifard et al., 2015; Serhan, 2005). Mammalian brains were also shown to be invariably rich in DHA (Crawford et al., 2009). Thus, it is necessary to obtain a better understanding of the potential metabolic and health effects of DHA, uncoupled by a higher EPA content. Tuna, one of the most important sources of EPA and DHA, is characterized by higher DHA and less EPA in its oil (e.g., EPA:DHA = 4.2:19.8 [Castellano et al., 2011], 3:13 [Ninio et al., 2005]). In this study, not only general tuna oil (60 mg of EPA and 260 mg of DHA per gram of oil) but also its fractionated and concentrated product with a higher DHA content (60 mg of EPA and 340 mg of DHA per gram of oil) was employed to investigate the effect of high‐DHA tuna oil on the alteration of the gut microbiota and obesity in high‐fat diet mice. We aimed to provide evidence for the clinical therapeutic potential of the dietary administration of high‐DHA fish oil preparations.

## 2 MATERIALS AND METHODS

### 2.1 Preparation of high‐DHA tuna oil

High‐DHA tuna oil was prepared according to the method proposed in our previous report (Chen et al., 2019). The composition of fatty acids was detected by gas chromatography–mass spectrometry (GC–MS; Agilent 7890/M7‐80EI system with a VOCOL column [60 m × 0.32 mm]). The initial temperature of the oven program was set at 60°C, increased to 260°C at a rate of 5°C/min, and then was maintained at 260°C for 40 min. The gas flow rate was 50 ml/min. The temperature of the injector was maintained at 260°C. The detected mass ranged from 30 to 425 *m*/*z* (Lu et al., 2017; Satil et al., 2003). The content ratios of EPA and DHA in the general tuna oils and its fractionated and concentrated product were 60 mg of EPA and 260 mg of DHA per gram of oil, 60 mg of EPA and 340 mg of DHA per gram of oil, respectively.

### 2.2 Animals and experimental design

All experimental procedures and animal care were performed according to the Guide for the Care and Use of Laboratory Animals prepared by the Ningbo University Laboratory Animal Center (affiliated with the Zhejiang Laboratory Animal Common Service Platform), and all of the animal protocols were approved by the Ningbo University Laboratory Animal Center under permit number SYXK (ZHE 2008‐0110).

Thirty ICR mice (4–5‐week‐old males) with an average weight of 23.2 ± 2.1 g (purchased from Laboratory Animal Center of Zhejiang Province; SCXK [Zhejiang] 2014 ± 0001) experienced 1 week of acclimatization with a standard chow diet (MD 17121; Mediscience Co., Ltd) and were randomly divided into five groups (six mice per group). Next, each group was housed in two separate cages (three mice per cage) under controlled conditions (23 ± 1°C; 12:12 hr light/dark cycle; 60 ± 5% relative humidity) with free access to food and water for 6 weeks. The five groups were as follows: (a) control group (Control), normal chow feeding (protein with 20% kcal, carbohydrate with 70% kcal, fat with 10% kcal; purchased from Laboratory Animal Center of Ningbo University, Ningbo, China) and received 200 ml of saline per day by gavage; (b) high‐fat diet group (HFD), high‐fat diet feeding (protein with 20% kcal, carbohydrate with 35% kcal, fat with 45% kcal purchased from Laboratory Animal Center of Ningbo University); (c) positive‐control drug group (Zocord), high‐fat diet feeding and received 3 mg kg^−1^ day^−1^ of Zocord by gavage; (d) high‐DHA tuna oil group (HDTO), high‐fat diet feeding and received 260 mg kg^−1^ day^−1^ of high‐DHA tuna oil by gavage; (e) conventional tuna oil group (TO), high‐fat diet feeding and received 260 mg kg^−1^ day^−1^ of conventional tuna oil by gavage.

The body weight of the mice was monitored every 5 days. At the end of 6 weeks, stool samples were collected, immediately immersed in liquid nitrogen and stored at −80°C for later microbiota analysis. After 12 hr of food deprivation, the fasting body weight was examined and all the mice were anesthetized with ether. Blood was collected from the orbital plexus, and the serum was further isolated by centrifugation at 1500 *g* at 4°C for 15 min and then stored at −80°C for subsequent biochemical testing. All the animals were sacrificed. Visceral tissues, including adipose (epididymal, subcutaneous, visceral, and interscapular) and liver tissues were dissected, weighed, instantly immersed in liquid nitrogen, and then stored at −80°C for further analysis.

### 2.3 Biochemical analysis

Liver samples were prepared by homogenization and centrifugation. Total cholesterol (TC), triglyceride (TG), HDL‐cholesterol (HDL‐C), and LDL‐cholesterol (LDL‐C) levels in the serum samples and liver samples were measured using commercial enzymatic kits purchased from Nanjing Jiancheng Bioengineering Institute according to the manufacturer's instructions.

Liverindex=[liverwetweight(g)/bodyweight(g)]×100%

### 2.4 Real‐time qPCR for the expression of genes related to obesity

The livers were homogenized in liquid nitrogen. RNA extraction was performed according to the RNA extraction kit instructions. The RNA was reverse transcribed into cDNA using a high‐capacity cDNA reverse transcription kit (Applied Biosystems, Life Technologies). qRT–PCR was performed using SYBR Green Master Mix and Quant Studio 6 Flex (Thermo Fisher Scientific Inc.) according to the manufacturer's instructions. The quantification of RNA samples was carried out using the Nanodrop 2000C system (Thermo Fisher Scientific Inc.). All the samples were run in duplicate on a single plate, and relative quantification was detected using the 2^−ΔΔCt^ method. The qRT–PCR primers used in this study are presented in Table 1. β‐Actin was used as an internal control.

**Table 1:** Primer sequences used in real‐time PCR analysis

Genes	Primer sequences	
	Forward primer (5′→3′)	Reverse primer (5′→3′)
β‐ACTIN	GAGAGGGAAATCGTGCGTGA	CTTCTCCAGGGAGGAAGAGGAT
ACC	GACAACACCTGTGTGGTGGA	AGGTTGGAGGCAAAGGACATT
FAS	CACAGCCCTGGAGAACTTGT	CGGTGGCTGTGTATTCCAGT
HMGCR	CTGATCCCCTTTGGCTCTTTCA	AGGCCGATGGTATACTTTCCAG
CPT‐1	AACCTTGGCTGCGGTAAGACTA	AGTGGGACATTCCTCTCTCAGG
SREBP‐2	ACAAGTCTGGCGTTCTGAGG	GATGCCCTTCAGGAGCTTGT
IL‐6	ACAAGTCCGGAGAGGAGACT	CAGGTCTGTTGGGAGTGGTATC

### 2.5 DNA extraction, 16S rRNA sequencing, and bioinformatic analysis

DNA from different samples was extracted using the E.Z.N.A. ®Stool DNA Kit (D4015; Omega, Inc.) according to the manufacturer's instructions, and the quantification of genomic DNA was executed using the Thermo NanoDrop 2000C system. The V3‐V4 region of the 16S rRNA gene was amplified using primers 338F (5′‐ACTCCTACGGGAGGCAGCAG‐3′) and 806R (5′‐GGACTACHVG GGTWTCTAAT‐3′). The 5′ ends of the primers were tagged with specific barcodes per sample and universal sequencing primers. The cycling and reaction conditions were 98°C for 30 s followed by 35 cycles of denaturation at 98°C for 10 s, annealing at 54°C/52°C for 30 s, and extension at 72°C for 45 s and then a final extension at 72°C for 10 min. The PCR products were confirmed by 2% agarose gel electrophoresis, and they were purified using AMPure XT beads (Beckman Coulter Genomics) and quantified by Qubit (Invitrogen).

Samples were sequenced using the Illumina MiSeq platform according to the manufacturer's recommendations provided by LC‐Bio. Quality filtering on the raw tags was performed under specific filtering conditions to obtain high‐quality clean tags according to FastQC (V 0.10.1). Chimeric sequences were filtered using Verseach software (v2.3.4). Sequences with ≥97% similarity were assigned to the same operational taxonomic units (OTUs) by Verseach (v2.3.4). Representative sequences were chosen for each OTU, and taxonomic data were then assigned to each representative sequence using the RDP (Ribosomal Database Project) classifier. Alpha diversity and beta diversity analysis were performed using QIIME (Version 1.8.0). Linear discriminant analysis (LDA) scores derived from the LDA effect size (LEfSe, https://huttenhower.sph.harvard.edu/galaxy/root?tool_id=lefse_upload) was executed to identify the specific bacteria (*p* < .05 and LDA score of >6.0; Segata et al., 2011). The correlations between the relative abundance of the key species and related metabolic indices and genes were conducted by Spearman's correlation in R software (version 3.6.2). The prediction of functional pathway variations in the gut microbiome at the OTU level was performed using the Tax4Fun R package (Aßhauer et al., 2015). Different analyses were conducted in STAMP (Parks et al., 2014), and Welch's *t* test was used for the comparison of two groups.

### 2.6 Statistical analysis

The data are expressed as the means ± *SEM* (standard error of the mean) and were analyzed using SPSS 23.0 statistics and OriginPro software. Differences between two groups were assessed using unpaired two‐tailed Student's *t* tests. Repeated measures one‐way analysis of variance (ANOVA) and Tukey's post hoc test (SPSS) were used. For the data whose distribution did not conform to the Gaussian model of heterogeneity, nonparametric Kruskal–Wallis analysis was conducted. Differences were considered statistically significant at *p* < .05.

## 3 RESULTS

### 3.1 HDTO and TO improve the features of obesity in high‐fat diet‐fed mice

**Figure 1:**
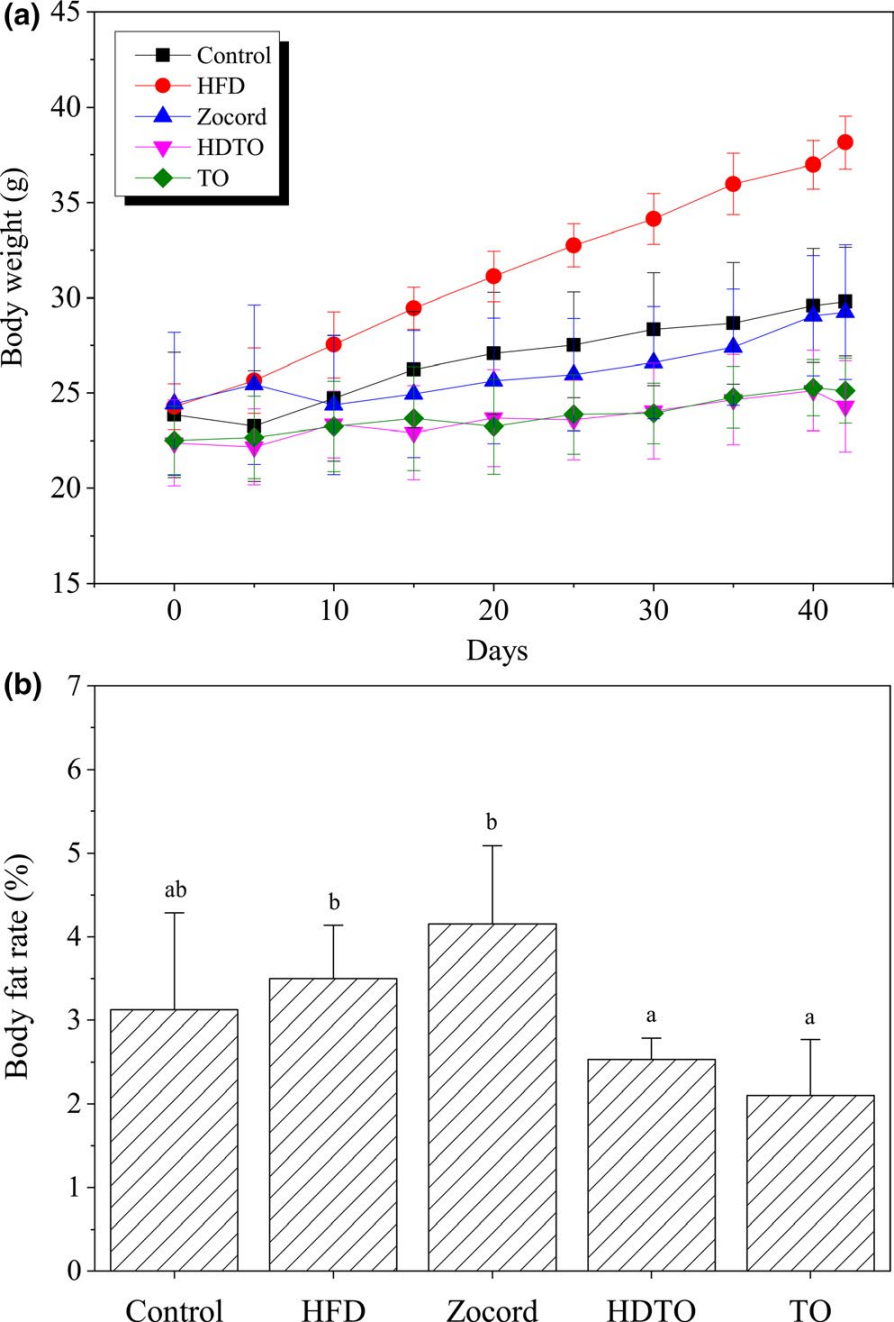
HDTO and TO prevent body weight gain and fat accumulation in HFD‐fed mice. (a) Variation in the body weight. (b) Body fat rate. Different letters correspond to statistically significant differences (*p* < .05) between groups. The values are expressed as the means ± *SEM*, *n* = 6

Four groups of mice were, respectively, provided with HFD and were administered 260 mg kg^−1^ day^−1^ of HDTO or TO, as well as Zocord, to identify the impact of HDTO and TO on the development of obesity. The positive‐control drug Zocord served as a treatment reference. As illustrated in Figure 1a, the mice that consumed a high‐fat diet gained more weight than the other groups from the 10th day, reaching a twofold increase at the final feeding trial compared with the control. In the Zocord group, the body weight was significantly decreased, but the body fat was higher than that in the HFD group (Figure 1b). Notably, two types of tuna oil could attenuate the body gain and reduce fat build‐up, and the suppressing effect was significantly better than that in Zocord treatment. However, the suppressing effect between HDTO and TO on the body weight and body fat rate showed no significant difference.

**Figure 2:**
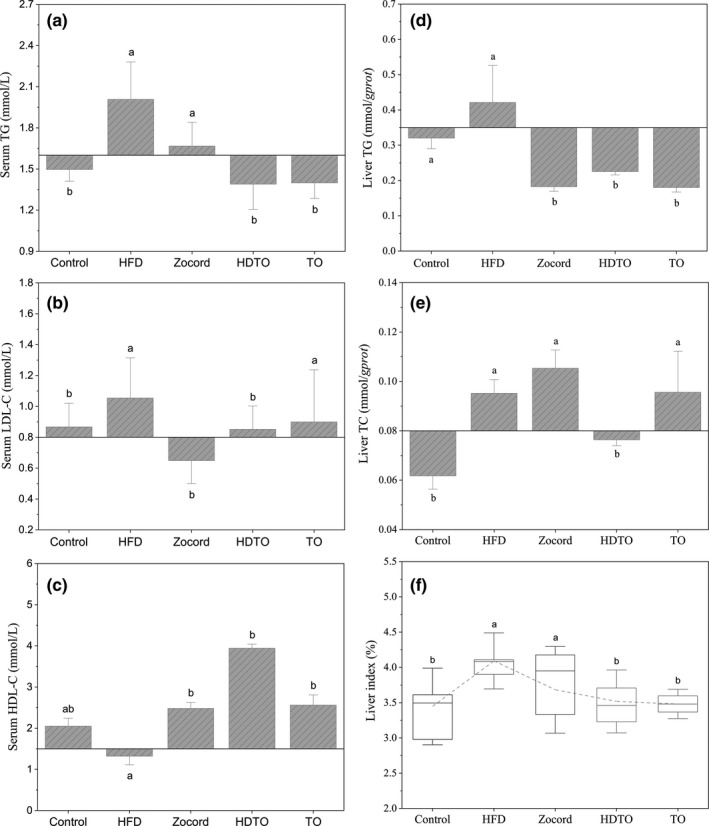
HDTO and TO administration improves lipid metabolism in HFD‐fed mice. Concentrations of serum (a) TG, (b) LDL‐C, (c) HDL‐C and liver (d) TG, (e) TC in mice, (f) liver index of the mice. Different letters correspond to statistically significant differences (*p* < .05) between groups. The values are expressed as the means ± *SEM*, *n* = 6

Compared with the control, 6‐week high‐fat diet feeding led to boosted levels of TG and LDL‐C and decreased levels of HDL‐C in the serum (Figure 2a–c). Similarly, the levels of TC and TG in the liver of the HFD groups were higher than those in the control group. Furthermore, HDTO and TO supplementation significantly decreased the serum TG and increased the serum HDL‐C levels in the mice fed a high‐fat diet.

Additionally, the level of serum LDL‐C was decreased due to HDTO and TO supplementation, and *p* < .05 compared with the HFD groups. The effect of Zocord supplementation was similar to that of the tuna oil. Notably, HDTO and TO also reduced the liver weights of the HFD‐fed mice to restore to the control level (Figure 2f). Regarding the concentrations of liver TG and TC, HDTO and TO supplementation led to decreased liver TG and TC levels (Figure 2d/e). Additionally, exceptions occurred for Zocord supplementation; the concentrations of liver TC were higher than those of mice fed a high‐fat diet. These results indicate that HDTO and TO can improve blood and liver metabolic parameters in obese mice, and the effect of HDTO treatment is more obvious.

### 3.2 HDTO and TO ameliorate the expression of lipid metabolism‐related genes and IL‐6 expression in high‐fat diet‐fed mice

**Figure 3:**
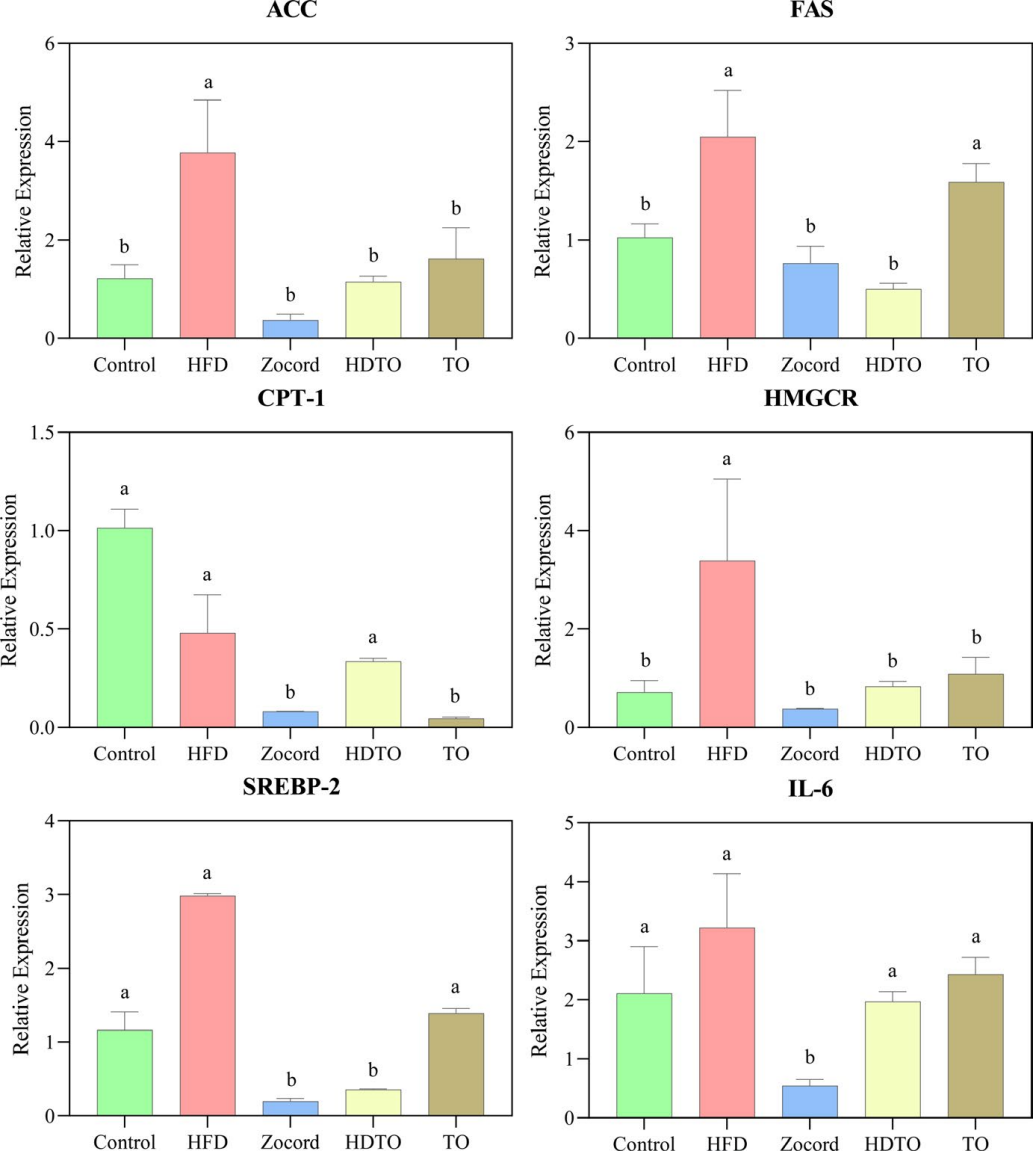
Effects of HDTO and TO supplementation on the relative expression of ACC, FAS, CPT‐1, HMGCR, SREBP‐2, and IL‐6 in the liver. The values are expressed as means ± *SEM*, and the different letters represent significant differences between different groups (*p* < .05)

The expression of genes involved in hepatic lipogenesis and lipolysis can be altered by HFD consumption. To confirm the reversal effect of high‐DHA tuna oil on these lipid metabolism‐related genes, the messenger RNA (mRNA) levels of fatty acid synthase (FAS), acetyl‐CoA carboxylase (ACC), carnitine palmitoyl transferase‐1 (CPT‐1), 3‐hydroxy‐3‐methyl glutaryl coenzyme A reductase (HMGCR), and sterol regulatory element‐binding protein‐2 (SREBP‐2) in the liver were investigated. As shown in Figure 3, a high‐fat diet led to the upregulation of ACC, FAS, HMGCR, and SREBP‐2, as well as the downregulation of CPT‐1, compared with the control group. In contrast to the HFD group, HDTO and TO supplementation significantly reduced the expression levels of ACC, FAS, HMGCR, and SREBP‐2 (*p* < .05). However, the reversal effect on the level of CPT‐1 did not occur. Furthermore, as reported in previous studies, HFD‐fed obese mice produced higher hepatic levels of pro‐inflammatory cytokines. In this study, interleukin‐6 (IL‐6) expression levels were notably elevated due to HFD induction compared with those in chow‐fed mice. Next, the expression pattern of this cytokine was reduced by HDTO and TO supplementation. Additionally, the reversal effects of Zocord administration were also observed in all genes. Overall, HDTO more significantly reversed the change triggered by HFD in mice.

### 3.3 HDTO and TO supplementation alters the structure of the gut microbiota in high‐fat diet‐fed mice

To elucidate the effects of dietary high‐DHA tuna oil on the gut microbiome in high‐fat diet‐induced obese mice, high‐throughput sequencing of 16S rRNA based on V3‐V4 hypervariable regions was used to analyze the variations in the gut microbial structure. The gut microbiota of the mice fed HFD + HDTO and HFD + TO was profiled and compared with that of the control group, HFD group and Zocord group. After double‐end splicing, quality control, and chimera filtering, 243,397 high‐quality sequencing reads were obtained from 15 fecal samples, and then the sequencing reads were clustered into OTUs at a 97% similar level.

**Figure 4:**
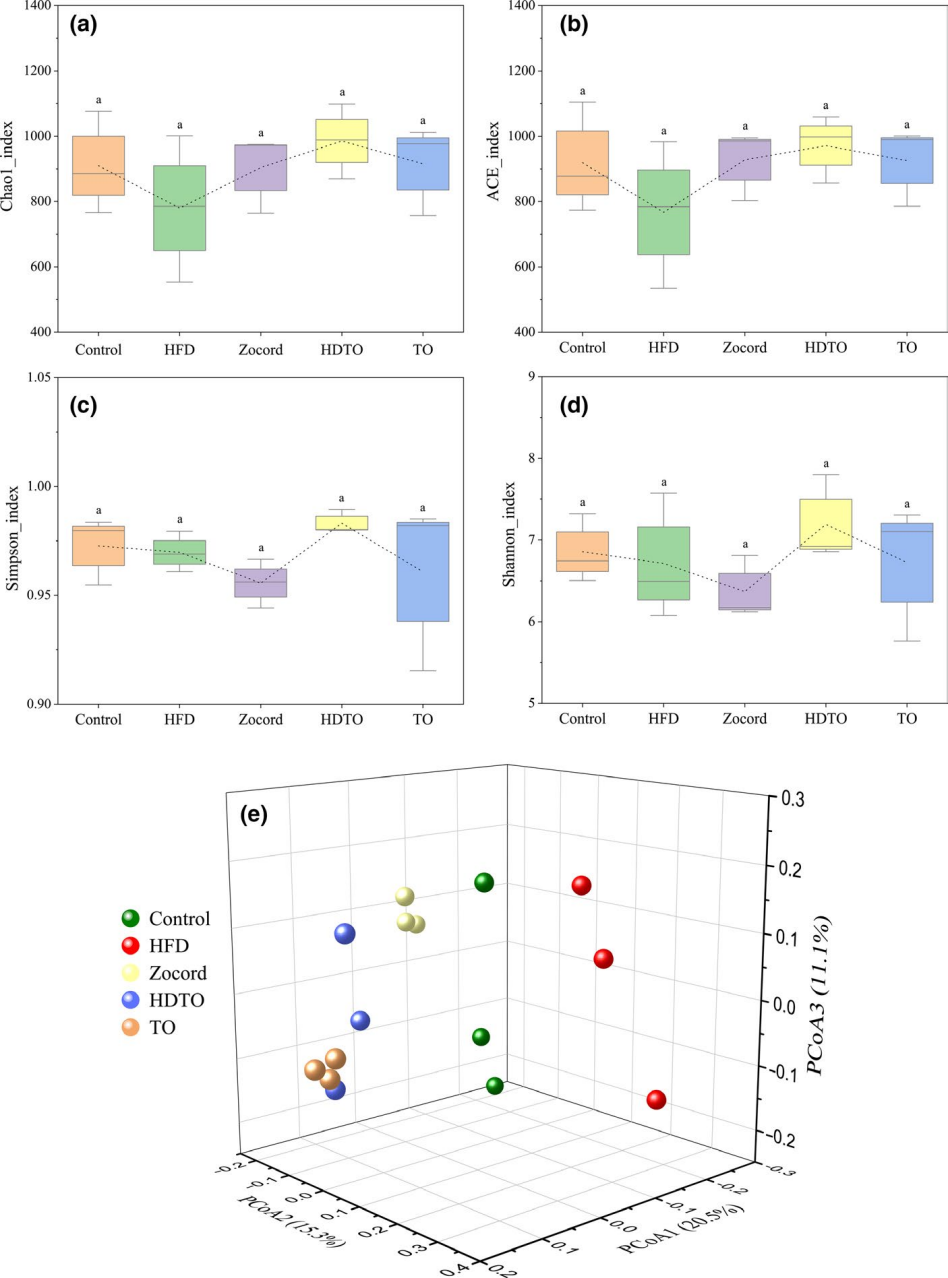
Alpha diversity analysis of (a) Chao1, (b) ACE, (c) Simpson, and (d) Shannon indices. (e) Principal coordinate analysis (PCoA) on the OTU level based on the unweighted Unifrac distance. The values are expressed as the means ± *SD*, and the different letters represent significant differences between different groups (*p* < .05)

Alpha diversity was applied in analyzing the complexity and diversity of species for samples using four parameters: two richness estimators (Chao1 and ACE) and two diversity indices (Shannon and Simpson). As suggested in Figure 4, HDTO and TO supplementation could prevent HFD‐induced reduction in microbial richness and diversity to some extent, especially HDTO treatment, but not to a statistically significant level (*p* > .05, Figure 4a–d). The effect of Zocord administration was positive on richness but negative on diversity compared with the HFD group.

The relationships among the gut microbiota communities of the groups were evaluated by unweighted Unifrac‐based PCoA at the OTU level. The results showed that the gut microbiome in the mouse was changed by a dietary high‐fat diet and distinctly diverged from the control groups. The gut microbiota of the HDTO, TO, and Zocord groups were all changed individually, and they clustered separately from the HFD group. Additionally, from the perspective of spatial distribution, HDTO and TO were closer to the control. This obvious clustering of the microbiota composition and varied distance in different groups suggest that HDTO and TO supplementation might revert the HFD‐induced gut microbiota composition to a normal status.

**Figure 5:**
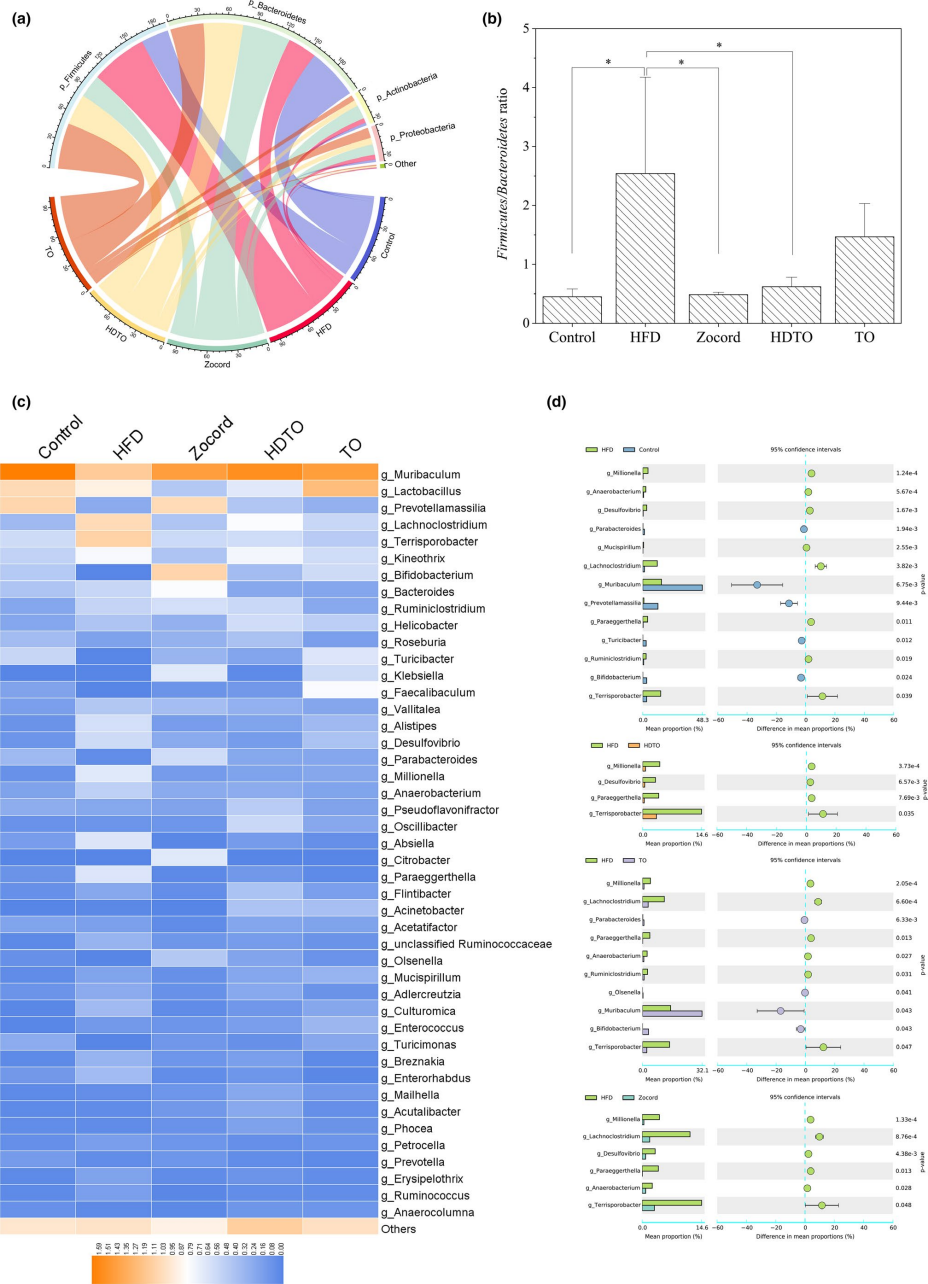
Changes in the gut microbiota related to HDTO and TO supplementation. (a) Relative abundance of the bacterial profile at the phylum level. (b) Firmicutes/Bacteroidetes (F/B) ratio. (c) Heatmap of the relative abundance of the bacterial profile at the genus level. (d) Mean proportions of the significantly different genera in mice between different groups (Welch's *t* test was performed to assess the difference between two groups). The data are shown as the means ± *SEM*, *p < .05

Specific changes in the gut microbiota related to HDTO and TO supplementation were further illuminated by the relative abundance of the bacterial profile at the phylum and genus levels. As indicated in Figure 5a, Firmicutes and Bacteroidetes were the dominant composition of gut microbiota in the mice, their total abundance reached approximately 90%. Notably, the relative abundance of Firmicutes in the HFD group was significantly increased compared with that in the control group (*p* = .001). However, this status was reversed by tuna oil supplementation, and dietary HDTO exhibited a more pronounced effect (*p* = .003 vs. the HFD group). Additionally, with the significant reduced abundance of *Firmicutes* in the HDTO and TO groups, the relative abundance of *Bacteroidetes* significantly increased compared with that in the high‐fat diet feeding group (*p* = .01). Thus, HDTO and TO supplementation could fully hamper the HFD‐triggered increases in the *Firmicutes*/*Bacteroidetes* (F/B) ratio (Figure 5b, *p* < .05), and restore the abundance to a normal level.

At the genus level, the dominant bacteria in the HDTO, TO, and control groups were relatively similar, and dietary HFD resulted in variation of the microbiota profile that was markedly different from that in the control group (Figure 5c). The relative abundances of the genera *Anaerobacterium*, *Desulfovibrio, Lachnoclostridium*, *Millionella*, *Mucispirillum*, *Paraeggerthella*, *Ruminiclostridium,* and *Terrisporobacter* were significantly upregulated in HFD compared with those in the control group, whereas the relative abundances of the genera *Bifidobacterium*, *Muribaculum*, *Parabacteroides*, *Prevotellamassilia,* and *Turicibacter* were markedly downregulated (Figure 5d).

Nevertheless, most of the genera determined in the stool have similar levels of relative abundance in the HDTO and TO groups to those in the control group (Figure 5c). HDTO supplementation significantly decreased the relative abundances of *Millionella*, *Desulfovibrio*, *Paraeggerthella*, and *Terrisporobacter* and restored them to similar levels as those in the control. However, TO supplementation significantly increased the relative abundances of *Parabacteroides*, *Muribaculum*, *Bifidobacterium*, and *Olsenella*, and notably decreased the relative abundances of *Millionella*, *Lachnoclostridium*, *Paraeggerthella*, *Anaerobacterium*, *Ruminiclostridium*, and *Terrisporobacter*. Additionally, Zocord administration reversed the increase in the abundances of *Millionella*, *Lachnoclostridium*, *Desulfovibrio*, *Paraeggerthella*, *Anaerobacterium*, and *Terrisporobacter* induced by dietary high‐fat diet. A significant decrease in the genus *Absiella* was also observed in the Zocord group.

### 3.4 Pivotal phylotypes of gut microbiota corresponding to HDTO and TO supplementation and their correlation with obesity‐related metabolism parameters

**Figure 6:**
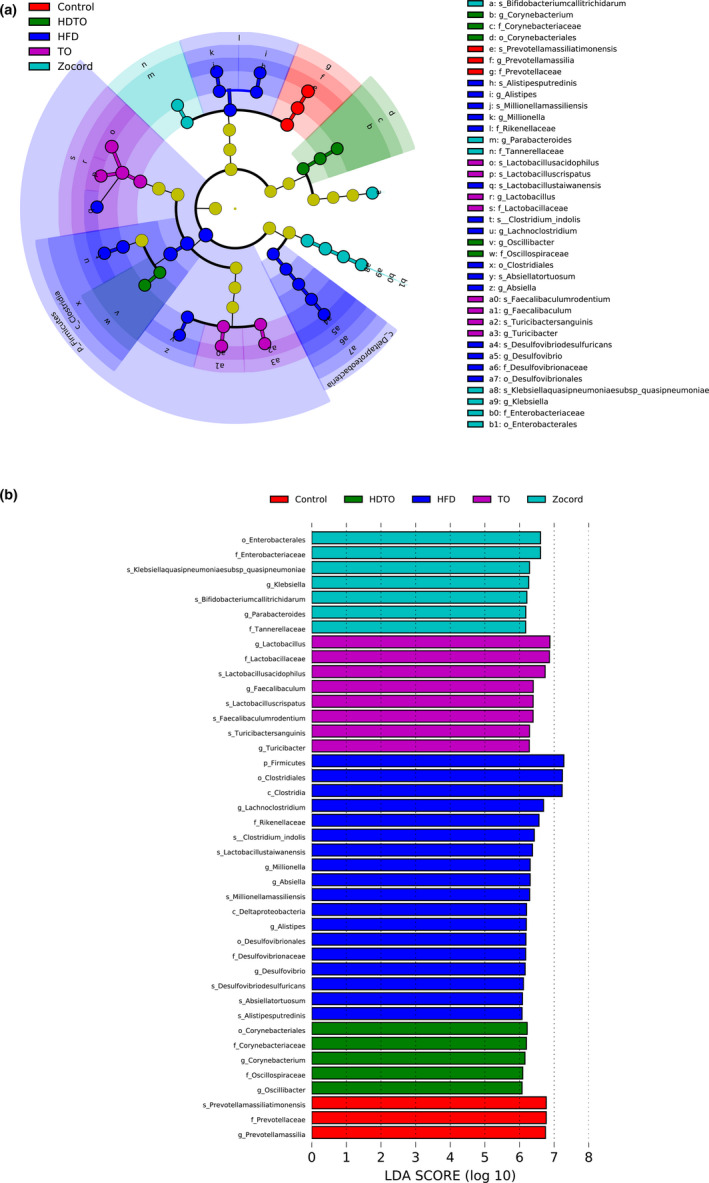
Key phylotypes of gut microbiota corresponding to HDTO and TO supplementation. LDA effect size (LEfSe) was performed to identify the different abundant taxa (LDA score was 6.0). (a) The cladograms show the most differently abundant taxa enriched in the microbiota. (b) The histograms show the LDA scores calculated for characteristics

The LDA effect size (LEfSe) method was employed to analyze the 16s rRNA sequencing data to discern the specific altered bacterial phenotypes and biomarkers of HFD and the dietary intervention groups (Figure 6). The results suggest that the specific bacterial taxa identified from the phylum to the genus level were different for each group. HDTO or TO contributed fewer different abundant features in mice fed a high‐fat diet plus tuna oil than only high‐fat diet‐fed mice. The phylum *Firmicutes* was the most important biomarker of HFD mice, showing a similar tendency to the results of analyses above. Furthermore, seven taxa that could represent the characteristics of dietary high‐fat diet all belonged to *Firmicutes*, including *Clostridia*, *Clostridiales*, *Lachnoclostridium*, *Absiella*, *Clostridium_indolis*, *Absiellatortuosum,* and *Lactobacillustaiwanensis*. The HFD group was also characterized by a high number of the class *Deltaproteobacteria*, order *Desulfovibrionales*, family *Desulfovibrionaceae*, *Rikenellaceae*, genus *Desulfovibrio*, *Alistipes*, *Millionella* and species *Desulfovibrio desulfuricans*, *Alistipes putredinis*, *Millionella massiliensis*. Regarding the HDTO group, the bacteria were enriched in order *Corynebacteriales*, family *Corynebacteriaceae*, genus *Corynebacterium*, and family *Oscillospiraceae*, genus *Oscillibacter*. Regarding the TO group, the bacteria were enriched in the family *Lactobacillaceae*, genus *Turicibacter*, *Faecalibaculum*, *Lactobacillus*, and species *Faecalibaculum rodentium*, *Lactobacillus acidophilus*, *Lactobacillus crispatus*, *Turicibacter sanguinis*. Additionally, the Zocord group was characterized by the relatively high abundance of the order *Enterobacterales*, family *Enterobacteriaceae* and *Tannerellaceae*, genus *Parabacteroides* and *Klebsiella*, and species *Bifidobacterium callitrichidarum* and *Klebsiellaquasipneumoniaesubsp_quasipneumoniae*.

**Figure 7:**
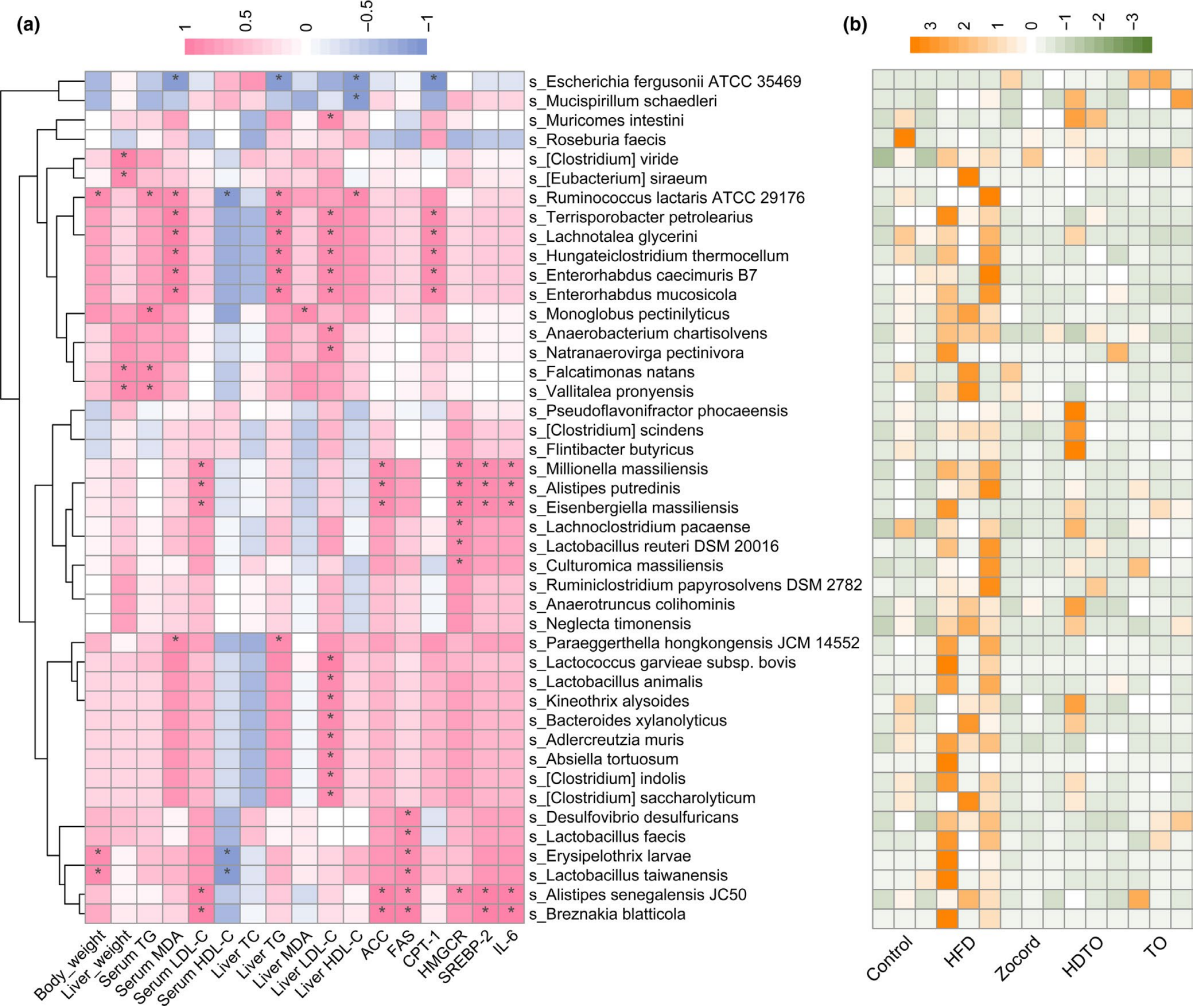
Heatmap of the biomarker of HFD at the species level (a) and (b) the correlation between the metabolic parameters and biomarkers determined by Spearman's correlation coefficient. The color blue indicates a negative correlation, whereas red shows a positive correlation. The significance levels are represented by * (*p* < .05)

The biomarkers at the species level in HFD‐fed mice were identified using LDA scores. Compared with the control group, the HFD group increased the relative abundances of 44 species in total, in which 32 species belonged to *Firmicutes*, four species belonged to *Actinobacteria*, five species belonged to *Bacteroidetes*, one species belonged to *Deferribacteres*, and two species belonged to *Proteobacteria*. HDTO and TO supplementation effectively reversed most of them toward the control level (Figure 7b). Furthermore, the potential correlations among these significantly changed taxa in the gut microbiome and obesity‐associated metabolism parameters were determined using Spearman's correlation analysis. As illustrated in Figure 7a, *Clostridium_viride* and *Eubacterium_siraeum*, *Anaerobacterium chartisolvens*, *Muricomes intestini* and *Natranaerovirga pectinivora* were positively related to liver weight and liver LDL‐C, respectively. Five species, including *Terrisporobacter petrolearius*, *Lachnotalea glycerini*, *Hungateiclostridium thermocellum*, *Enterorhabdus caecimuris B7*, and *Enterorhabdus mucosicola*, were also positively correlated with the expression levels of serum MDA, liver TG and LDL‐C, and the CPT‐1 gene. *Falcatimonas natans* and *Vallitalea pronyensis* were positively related to liver weight and serum TG. *Millionella massiliensis*, *Alistipes putredinis*, and *Eisenbergiella massiliensis* were positively correlated with the expression levels of serum LDL‐C, and ACC, HMGCR, SREBP‐2, and IL‐6 genes in the liver. The three species *Lachnoclostridium pacaense*, *Lactobacillus reuteri DSM 20016,* and *Culturomica massiliensis* were significantly related to HMGCR gene expression in the liver. The eight species *Lactococcus garvieae* subsp. *bovis*, *Lactobacillus animalis*, *Kineothrix alysoides*, *Bacteroides xylanolyticus*, *Adlercreutzia muris*, *Absiella tortuosum*, *Clostridium_indolis*, and *Clostridium_saccharolyticum* showed a positive correction with liver LDL‐C. *Desulfovibrio desulfuricans* and *Lactobacillus faecis* were positively associated with the FAS gene in the liver. *Erysipelothrix larvae* and *Lactobacillus taiwanensis* were positively and negatively associated with body weight, FAS, and serum HDL‐C, respectively. *Alistipes senegalensis JC50* was positively related to six parameters—serum LDL‐C and the ACC, FAS, HMGCR, SREBP‐2, and IL‐6 genes in the liver. The parameters positively related to *Breznakia blatticola* were LDL‐C and the ACC, FAS, SREBP‐2, and IL‐6 genes in the liver. *Monoglobus pectinilyticus* was positively related to serum TG and liver MDA. *Ruminococcus lactaris ATCC 29176* was positively correlated with body weight, serum TG and MDA, and liver TG and HDL‐C and was negatively correlated with serum HDL‐C. However, *Mucispirillum schaedleri* was negatively correlated with liver HDL‐C, and *Escherichia fergusonii ATCC 35469* was concurrently negatively correlated with serum MDA and TG, HDL‐C and CPT‐1 genes in the liver. The above correlation was significant at *p* < .05.

### 3.5 HDTO and TO supplementation alters the metabolic pathways

**Figure 8:**
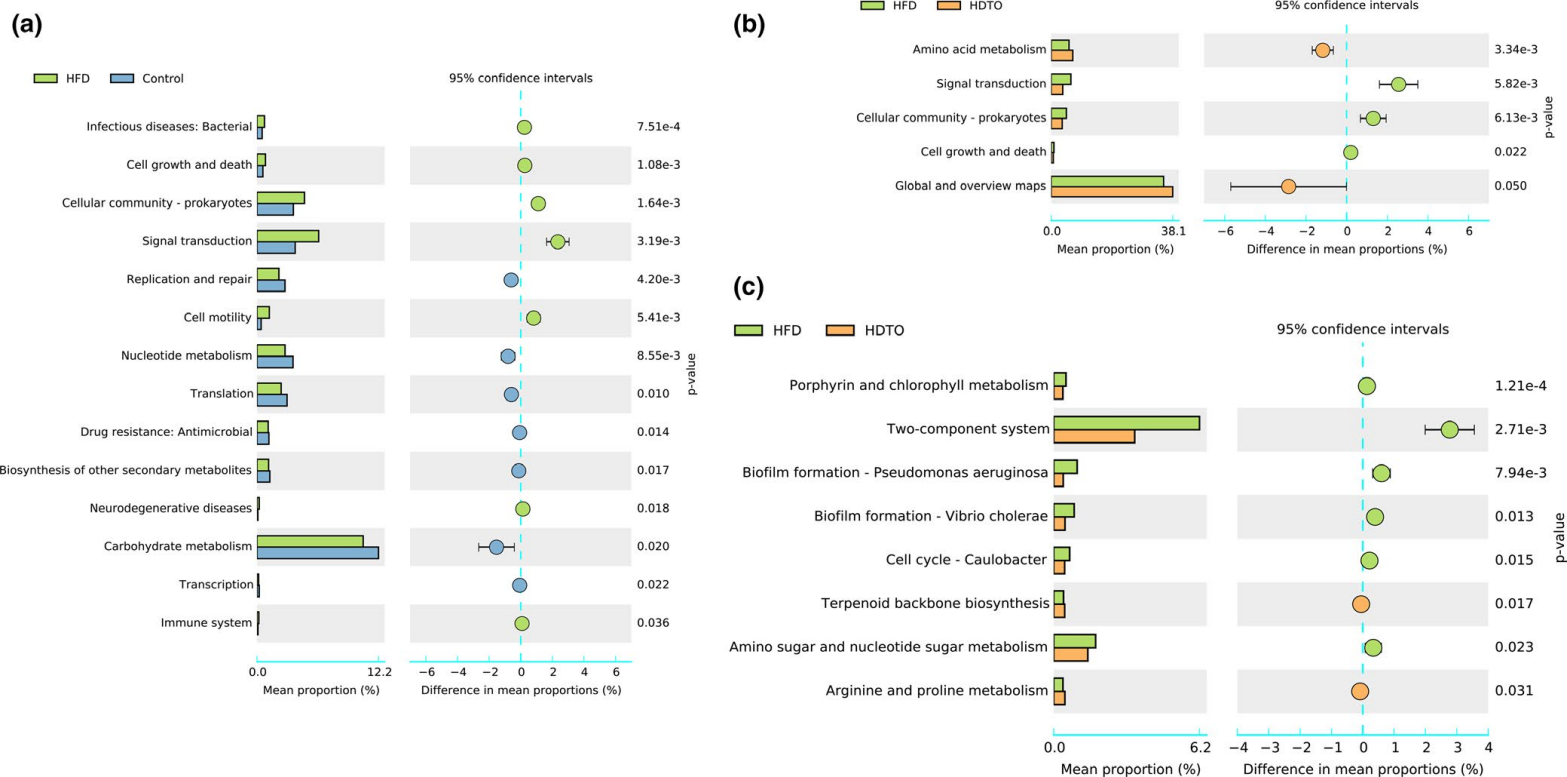
Predicted metabolic profile of the stool microbiome after HDTO and TO supplementation. The 16S rRNA data were further analyzed as indicated by Tax4Fun. Statistical significance difference between two groups based on Welch's *t* test in STAMP. (a) HFD versus Control at level II. (b) HFD versus HDTO at level II. (c) HFD versus HDTO at level III. The data are expressed as the means ± *SEM*

Because dietary HDTO resulted in the variation of the gut microbiota structure in high‐fat diet consumption mice, the functional profiles related to HDTO supplementation should be further predicted. This prediction was performed by Tax4Fun, and 46 metabolic pathways were predicted at level 2. HFD consumption significantly regulated 14 metabolic pathways (Figure 8a), a very impressive changing rate, in which the upregulated pathways included infectious diseases: bacterial, cell growth and death, cellular community‐prokaryotes, signal transduction, cell motility, neurodegenerative diseases and immune system; and the downregulated pathways were replication and repair, nucleotide metabolism, translation, drug resistance: Antimicrobial, biosynthesis of other secondary metabolites, carbohydrate metabolism, and transcription. Compared with the HFD group (Figure 8b,c), HDTO supplementation regulated five functional pathways. Cell growth and death, signal transduction and cellular community‐prokaryotes were effectively downregulated and restored to the abundance level of the control group. Interestingly, dietary HDTO upregulated the pathways of level 2 involved in amino acid metabolism and global and overview maps (metabolic pathways, biosynthesis of secondary metabolites, microbial metabolism in diverse environments, biosynthesis of antibiotics, carbon metabolism, 2‐oxocarboxylic acid metabolism, fatty acid metabolism, degradation of aromatic compounds, and biosynthesis of amino acids in level 3).

## 4 DISCUSSION

Obesity triggers many diseases, such as heart disease, dyslipidemia, cancer, and 2 type diabetes. In recent years, accumulating studies in animals or humans showed that dietary supplementation with n‐3 PUFAs is a potentially feasible nutritional strategy to prevent obesity. Thus, many studies to date have exhibited positive effects of supplementation with fish oil containing EPA and DHA on obesity. However, the fish oils used in these studies were commercially available fish oil with a higher EPA content (Molinar‐Toribio et al., 2015; Parker et al., 2019). Additional evidence has indicated the role of the ratios of DHA and EPA in the prevention and treatment of chronic disease in rat models (Liu et al., 2018; Molinar‐Toribio et al., 2015). Furthermore, a study (Cottin et al., 2011) explored the known differential effects of EPA and DHA in human subjects and concluded that there is an evident potency of DHA to improve several cardiovascular risk factors. Thus, fish oils with a higher content of DHA than EPA might have different health benefits compared with the high‐EPA fish oil traditionally used. In this study, to fill this knowledge gap and extend the work of the currently available literature, tuna oil with an EPA/DHA ratio of 6:26 and its fractionated and concentrated oil (EPA:DHA = 6:34) were employed to gain a better understanding of the potential effects on obesity mitigation and gut microbiota in HFD mice.

As presented in this study, HDTO and TO supplementation effectively attenuated the features of obesity in high‐fat diet‐fed mice. The weight gain, liver weight, and body fat rate of the mice were significantly reduced accompanied by HDTO and TO consumption. Deterioration induced by HFD in most of the levels of lipid metabolism parameters in the serum and liver—TC, TG, LDL‐C, and HDL‐C—could be suppressed and even restored to the control level. Hence, regarding the variation in these parameters, HDTO and TO could improve the obesity feature in obese mice, and the overall treatment effect of HDTO was better than that of TO.

These improvements could be further demonstrated by the amelioration of lipid metabolism‐related gene expression in high‐fat diet‐fed mice. Often, obesity is associated with lipid metabolism disorders. The liver is the center of fat metabolism, and, together with the gallbladder, it can achieve fat digestion. However, in the case of metabolic disorders, the above process cannot be carried out smoothly. Transfats that are not digested will be accumulated and produced toxins, which not only affect the function of liver detoxification but also causes obesity, hyperlipidemia, or fatty liver. What factors cause these troubles? Accumulated evidence has indicated that hepatic gene expression involved in lipid metabolism can be altered by HFD‐induced obesity. ACC is a rate‐limiting enzyme for fatty acid synthesis, whose increased gene expression can promote fat synthesis; FAS can provide one storified fatty acid substrate for triacylglycerol to result in the enhancement of fatty acid synthesis and accumulation of TG; HMGCR catalyzes the diminution of HMG‐CoA to CoA and mevalonate, which is the rate restrictive reaction in the de novo synthesis of cholesterol; SREBP‐2 is the prominent isoform supporting cholesterol synthesis and uptake; CPT‐1 is the rate‐limiting enzyme of fatty acid β‐oxidation. The results of this study revealed that HDTO and TO intervention could significantly reverse the changes in the hepatic mRNA expression levels of ACC, FAS, HMGCR, SREBP‐2, and CPT‐1. Therefore, HDTO and TO might prevent body weight gain, fat accumulation, and increase lipid levels by suppressing adipogenic gene expression. Furthermore, considering the predicted metabolic pathways changed by HDTO and TO supplementation, the pathway of global and overview maps involved in metabolic pathways, biosynthesis of secondary metabolites, microbial metabolism in diverse environments, carbon metabolism, 2‐oxocarboxylic acid metabolism, and fatty acid metabolism was only upregulated by HDTO supplementation. Thus, we might infer that the reversed effect of the expression level of the ACC, FAS, HMGCR, SREBP‐2, and CPT‐1 genes of HDTO supplementation was better than that in TO was due to the upregulation of this pathway. Generally, obesity is characterized by a low‐grade of inflammation, and IL‐6 concentrations are often mildly elevated in obesity. The elevated mRNA expression levels of the pro‐inflammatory molecule IL‐6 in tuna oil‐treated groups indicated the anti‐inflammatory effect of HDTO and TO.

Gut dysbiosis is a critical factor in the development of obesity and metabolic syndrome. The modulation of gut microbiota has become a promising pharmacological approach in the prevention of various chronic diseases. According to previous reports, an increased richness in the gut microbial diversity is negatively correlated with obesity and various disease states (Ji et al., 2019; Sánchez et al., 2017), and obesity triggers an increase in the relative abundance of *Bacteroidetes* and a decrease in the relative abundance of *Firmicutes*. Obesity can be marked by this increased proportion of F/B. However, growing evidence has indicated that the gut microbiota composition and structure can be reshaped by the interaction between dietary components and intestinal microorganisms (Wang et al., 2019). HDTO and TO supplementation increase the alpha diversity of gut microbiota in HFD mice; PCoA analyses revealed a significant separation of microbiota communities between the HFD group and the two groups treated with HDTO and TO. Additionally, HDTO supplementation resulted in a farther reduction in the F/B ratio. *Bacteroidetes* and *Firmicutes* are two main communities that affect energy metabolism homeostasis (Xu et al., 2017). A lower F/B ratio often reflects less energy extraction from the diet, and the interventions that prevented obesity in animals and humans are potent (Sasaki et al., 2013; Turnbaugh et al., 2006).

HDTO and TO treatment could all mitigate the outgrowth of harmful bacteria (*Desulfovibrio*, *Paraeggerthella*, and *Terrisporobacter*) caused by HFD. At the same time, TO treatment could also effectively suppress the increase in *Millionella*, *Lachnoclostridium*, *Anaerobacterium*, and *Ruminiclostridium*, which were reported to be potentially related to diet‐induced obesity. Furthermore, the genera *Parabacteroides* and *Muribaculum* were reported to be negatively correlated with obesity phenotypes. *Bifidobacterium*, a well‐known beneficial bacterium responsible for oligosaccharide metabolism, was significantly elevated in relative abundance by TO treatment. Zocord administration had the similar suppressing effect on the above obesity‐related bacteria. Therefore, these effects of inhibiting harmful bacteria and blooming beneficial bacteria by HDTO and TO treatment might contribute to the prevention of microbial dysbiosis caused by HFD.

Intestinal bacteria influence mammalian physiology and contribute to nutrient acquisition, inflammatory reactions, energy harvest and lipid metabolism, and they are closely related to obesity and metabolic diseases (Frazier et al., 2011; Kasubuchi et al., 2015). At the species level, as a potential diagnostic biomarker of dysbiosis, the increased relative abundance of *Desulfovibrio desulfuricans* was associated with metabolic disorders and related inflammation (Sun et al., 2015; Weglarz et al., 2003). The *Desulfovibrio* genus is known to include a sulfate‐reducing bacterium that can metabolize sulfate to produce hydrogen sulfide. The latter in the intestinal tract inhibits the metabolic pathway of intestinal epithelial cells using butyric acid, damages the intestinal epithelial mucosa, and induces chronic inflammation (Pitcher & Cummings, 1996). According to our study, the relative abundance of this species was also positively correlated with FAS gene expression. Adipose FAS mRNA expression is significantly associated with obesity, predominantly visceral fat accumulation, impaired insulin sensitivity, and circulating adipokines (Berndt et al., 2007). However, the increased prevalence of this species by a high‐fat diet was significantly attenuated by HDTO and TO administration, indicating the potential effects of HDTO and TO in the prevention of obesity and related metabolic disease. The other biomarker, *Alistipes putredinis*, belongs to the genus *Alistipes* and is positively related to the gene expression of FAS, HMGCR, SREBP‐2, and IL‐6 in the liver and serum LDL‐C. As reported previously, *Alistipes* is a harmful microorganism, and its abundance is positively correlated with some obesity‐related parameters, such as weight and serum TG and IL‐6 gene expression (Kang et al., 2019; Xu et al., 2015). These results indicate that the anti‐obesity regulation of HDTO and TO is not only associated with lipid absorption but also with inflammatory immunity. Compared with *Alistipes putredinis* and *Desulfovibrio desulfuricans*, knowledge about *Millionella massiliensis* is very sparse.

Similar to regulating taxonomic compositions, HDTO supplementation also modulated the functional profiling of intestinal microbial communities. HDTO feeding to HFD‐fed mice might alter the arginine and proline metabolism of intestinal microbiota. Previous studies have demonstrated the critical role of arginine and proline in regulating gut mucosal homeostasis and inflammation (Van de Velde et al., 2017; Xu et al., 2016). Several studies have also evaluated the relationship between obesity and arginine and proline metabolism. The serum levels of proline and arginine have also been found in obese and hyperlipidemic adults (Li et al., 2016; Newgard et al., 2009). The major metabolomic amino acid signature with the upregulation of arginine and proline was shown to be associated with obesity, insulin resistance, and lipid concentrations. L‐ornithine and hydroxyproline, two key components in the pathway of arginine and proline metabolism, might be the vehicle of obesity (Moran‐Ramos et al., 2017).

## 5 CONCLUSION

In summary, high‐DHA tuna oil significantly ameliorated obesity and metabolic dysfunctions in mice fed a high‐fat diet, and these anti‐obesity effects might be mediated by gut microbiota. HDTO and TO markedly decreased the number of obesity‐promoting bacteria, *Desulfovibrio*, *Paraeggerthella*, *Terrisporobacter*, *Millionella*, *Lachnoclostridium*, *Anaerobacterium* and *Ruminiclostridium*, and restored the increase in the F/B ratio and specific regulation of community structure. Particularly, *Desulfovibrio desulfuricans*, *Alistipes putredinis*, and *Millionella massiliensis*, as potential diagnostic biomarkers of dysbiosis, were dramatically increased in relative abundances by HDTO treatment. Furthermore, according to the prediction, the functional pathway of HDTO supplementation‐regulated obesity might be arginine and proline metabolism in intestinal microbiota. Overall, the regulatory effect of HDTO occurred before that of TO. Tuna oil with a higher DHA content might be a promising therapeutic option in mitigating obesity. However, its quantitative usage and possible anti‐obesity mechanisms need to be further clarified. Further understanding of the mechanisms that underlie microbial resilience toward external perturbations will be a crucial requirement for microbiome‐directed precision treatment.

## AUTHOR CONTRIBUTIONS

X. Su designed this study. J. Zhang, J. Han, T. Ming, J. Zhou, C. Lu, and Y. Li carried on the animal experiments and finished the gut microbiota analysis. J. Zhang, J. Han, C. Lu, and X. Su prepared the manuscript.

## References

[fsn31941-bib-0001] Aßhauer, K. P. , Wemheuer, B. , Daniel, R. , & Meinicke, P. (2015). Tax4Fun: Predicting functional profiles from metagenomic 16S rRNA data. Bioinformatics, 31(17), 2882–2884. 10.1093/bioinformatics/btv287 25957349 PMC4547618

[fsn31941-bib-0002] Berndt, J. , Kovacs, P. , Ruschke, K. , Klöting, N. , Fasshauer, M. , Schön, M. R. , Körner, A. , Stumvoll, M. , & Blüher, M. (2007). Fatty acid synthase gene expression in human adipose tissue: Association with obesity and type 2 diabetes. Diabetologia, 50, 1472–1480. 10.1007/s00125-007-0689-x 17492427

[fsn31941-bib-0003] Cani, P. D. , Osto, M. , Geurts, L. , & Everard, A. (2012). Involvement of gut microbiota in the development of low‐grade inflammation and type 2 diabetes associated with obesity. Gut Microbes, 3(4), 279–288. 10.4161/gmic.19625 22572877 PMC3463487

[fsn31941-bib-0004] Castellano, C. A. , Audet, I. , Laforest, J. P. , Matte, J. J. , & Suh, M. (2011). Fish oil diets alter the phospholipid balance, fatty acid composition, and steroid hormone concentrations in testes of adult pigs. Theriogenology, 76(6), 1134–1145. 10.1016/j.theriogenology.2011.05.022 21752454

[fsn31941-bib-0005] Chen, Y. , Cheong, L. Z. , Zhao, J. H. , Panpipat, W. , Wang, Z. P. , Li, Y. , Lu, C. Y. , Zhou, J. , & Su, X. R. (2019). Lipase‐catalyzed selective enrichment of omega‐3 polyunsaturated fatty acids in acylglycerols of cod liver and linseed oils: Modeling the binding affinity of lipases and fatty acids. International Journal of Biological Macromolecules, 123, 261–268. 10.1016/j.ijbiomac.2018.11.049 30423396

[fsn31941-bib-0006] Cottin, S. C. , Sanders, T. A. , & Hall, W. L. (2011). The differential effects of EPA and DHA on cardiovascular risk factors. Proceedings of the Nutrition Society, 70(2), 215–231. 10.1017/S0029665111000061 21349231

[fsn31941-bib-0007] Crawford, M. A. , Bazinet, R. P. , & Sinclair, A. J. (2009). Fat intake and CNS functioning: Ageing and disease. Annals of Nutrition and Metabolism, 55, 202–228. 10.1159/000229003 19752543

[fsn31941-bib-0008] Crinò, A. , Fintini, D. , Bocchini, S. , & Grugni, G. (2018). Obesity management in prader‐willi syndrome: Current perspectives. Diabetes, Metabolic Syndrome and Obesity: Targets and Therapy, 11, 579–593. 10.2147/DMSO.S141352 PMC6175547 30323638

[fsn31941-bib-0009] da Cunha de Sá, R. D. C. , Crisma, A. R. , Cruz, M. M. , Martins, A. R. , Masi, L. N. , do‐Amaral, C. L. , Curi, R. , & Alonso‐Vale, M. I. C. (2016). Fish oil prevents changes induced by a high‐fat diet on metabolism and adipokine secretion in mice subcutaneous and visceral adipocytes. The Journal of Physiology, 594(21), 6301–6317. 10.1113/JP272541 27558442 PMC5088242

[fsn31941-bib-0010] Daniel, H. , Gholami, A. M. , Berry, D. , Desmarchelier, C. , Hahne, H. , Loh, G. , Mondot, S. , Lepage, P. , Rothballer, M. , Walker, A. , Böhm, C. , Wenning, M. , Wagner, M. , Blaut, M. , Schmitt‐Kopplin, P. , Kuster, B. , Haller, D. , & Clavel, T. (2014). High‐fat diet alters gut microbiota physiology in mice. The ISME Journal, 8, 295–308. 10.1038/ismej.2013.155 24030595 PMC3906816

[fsn31941-bib-0011] Fabbrini, E. , Sullivan, S. , & Klein, S. (2010). Obesity and nonalcoholic fatty liver disease: biochemical, metabolic, and clinical implications. Hepatology, 51, 679–689. 10.1002/hep.23280 20041406 PMC3575093

[fsn31941-bib-0012] Fard, S. G. , Wang, F. L. , Sinclair, A. J. , Elliott, G. , & Turchini, G. M. (2019). How does high DHA fish oil affect health? A systematic review of evidence. Critical Reviews in Food Science and Nutrition, 59(11), 1684–1727. 10.1080/10408398.2018.1425978 29494205

[fsn31941-bib-0013] Flachs, P. , Rossmeisl, M. , Bryhn, M. , & Kopecky, J. (2009). Cellular and molecular effects of n‐3 polyunsaturated fatty acids on adipose tissue biology and metabolism. Clinical Science, 116(1), 1–16. 10.1042/CS20070456 19037880

[fsn31941-bib-0014] Frazier, T. H. , DiBaise, J. K. , & McClain, C. J. (2011). Gut Microbiota, intestinal permeability, obesity‐induced inflammation, and liver injury. Journal of Parenteral and Enteral Nutrition, 35(5S), 14S–20S. 10.1177/0148607111413772 21807932

[fsn31941-bib-0015] Ghasemifard, S. , Hermon, K. , Turchini, G. M. , & Sinclair, A. J. (2015). Metabolic fate (absorption, b‐oxidation and deposition) of long‐chain n‐3 fatty acids is affected by sex and by the oil source (krill oil or fish oil) in the rat. British Journal of Nutrition, 114(5), 684–692. 10.1017/S0007114515002457 26234617

[fsn31941-bib-0016] Ikuo, K. , Kentaro, O. , Daisuke, I. , Takeshi, I. , Kumi, K. , Takeshi, M. , Kazuya, T. , Daiji, K. , Kanako, H. , Taeko, T. , Tomoyuki, T. , Satoshi, M. , Go, S. , Hiroshi, I. , & Gozoh, T. (2013). The gut microbiota suppresses insulin‐mediated fat accumulation via the short‐chain fatty acid receptor GPR43. Nature Communications, 4, 1829–1841. 10.1038/ncomms2852 PMC3674247 23652017

[fsn31941-bib-0017] Jérôme, B. , Sandrine, B. , Quentin, E. , Célia, B. , & Michel, N. (2019). N‐3 polyunsaturated fatty acids: An innovative strategy against obesity and related metabolic disorders, intestinal alteration and gut microbiota dysbiosis. Biochimie, 159, 66–71. 10.1016/j.biochi.2019.01.017 30690133

[fsn31941-bib-0018] Ji, Y. , Park, S. , Chung, Y. , Kim, B. , Park, H. , Huang, E. , Jeong, D. , Jung, H.‐Y. , Kim, B. , Hyun, C.‐K. , & Holzapfel, W. H. (2019). Amelioration of obesity‐related biomarkers by Lactobacillus sakei CJLS03 in a high‐fat diet‐induced obese murine model. Scientific Reports, 9, 6821 10.1038/s41598-019-43092-y 31048785 PMC6497927

[fsn31941-bib-0019] Kahn, R. (2008). Metabolic syndrome—what is the clinical usefulness? The Lancet, 371(9628), 1892–1893. 10.1016/S0140-6736(08)60731-X 18501420

[fsn31941-bib-0020] Kang, Y. B. , Li, Y. U. , Du, Y. H. , Guo, L. Q. , Chen, M. H. , Huang, X. W. , Yang, F. , Hong, J. A. , & Kong, X. Y. (2019). Konjaku flour reduces obesity in mice by modulating the composition of the gut microbiota. International Journal of Obesity, 43, 1631–1643. 10.1038/s41366-018-0187-x 30242233

[fsn31941-bib-0021] Kasubuchi, M. , Hasegawa, S. , Hiramatsu, T. , Ichimura, A. , & Kimura, I. (2015). Dietary gut microbial metabolites, short‐chain fatty acids, and host metabolic regulation. Nutrients, 7(4), 2839–2849. 10.3390/nu7042839 25875123 PMC4425176

[fsn31941-bib-0022] Lavanya, V. , & Rana, K. G. (2019). Contribution of adipogenesis to healthy adipose tissue expansion in obesity. The Journal of Clinical Investigation, 129(10), 4022–4031. 10.1172/JCI129191 31573549 PMC6763245

[fsn31941-bib-0023] Lee, M. J. , Wu, Y. , & Fried, S. K. (2013). Adipose tissue heterogeneity: Implication of depot differences in adipose tissue for obesity complications. Molecular Aspects of Medicine, 34(1), 1–11. 10.1016/j.mam.2012.10.001 23068073 PMC3549425

[fsn31941-bib-0024] Li, Q. , Gu, W. , Ma, X. , Liu, Y. , Jiang, L. , Feng, R. , & Liu, L. (2016). Amino acid and biogenic amine profile deviations in an oral glucose tolerance test: A comparison between healthy and hyperlipidaemia individuals based on targeted metabolomics. Nutrients, 8(6), 379–394. 10.3390/nu8060379 PMC4924220 27338465

[fsn31941-bib-0025] Liu, L. , Hu, Q. L. , Wu, H. H. , Wang, X. J. , Gao, C. , Chen, G. X. , Yao, P. , & Gong, Z. Y. (2018). Dietary DHA/EPA ratio changes fatty acid composition and attenuates diet‐induced accumulation of lipid in the liver of ApoE−/− mice. Oxidative Medicine and Cellular Longevity, 2018, 6256802 10.1155/2018/6256802 30538803 PMC6261399

[fsn31941-bib-0026] Lu, C. Y. , Sun, T. T. , Li, Y. Y. , Zhang, D. J. , Zhou, J. , & Su, X. R. (2017). Modulation of the gut microbiota by krill oil in mice fed a high‐sugar high‐fat diet. Frontiers in Microbiology, 8, 905–916. 10.3389/fmicb.2017.00905 28567037 PMC5434167

[fsn31941-bib-0027] Mayu, K. , Sae, H. , Takero, H. , Atsuhiko, I. , & Ikuo, K. (2015). Dietary gut microbial metabolites, short‐chain fatty acids, and host metabolic regulation. Nutrients, 7(4), 2839–2849. 10.3390/nu7042839 25875123 PMC4425176

[fsn31941-bib-0028] Molinar‐Toribio, E. , Pérez‐Jiménez, J. , Ramos‐Romero, S. , Romeu, M. , Giralt, M. , Taltavull, N. , Muñoz‐Cortes, M. , Jáuregui, O. , Méndez, L. , Medina, I. , & Torres, J. L. (2015). Effect of n‐3 PUFA supplementation at different EPA:DHA ratios on the spontaneously hypertensive obese rat model of the metabolic syndrome. British Journal of Nutrition, 113(6), 878–887. 10.1017/S0007114514004437 25720761

[fsn31941-bib-0029] Moran‐Ramos, S. , Ocampo‐Medina, E. , Gutierrez‐Aguilar, R. , Macias‐Kauffer, L. , Villamil‐Ramirez, H. , Lopez‐Contreras, B. E. , Leon‐Mimila, P. , Vega‐Badillo, J. , Gutierrez‐Vidal, R. , Villarruel‐Vazquez, R. , Serrano‐Carbajal, E. , Del‐Rio‐Navarro, B. E. , Huertas‐Vazquez, A. , Villarreal‐Molina, T. , Ibarra‐Gonzalez, I. , Vela‐Amieva, M. , Aguilar‐Salinas, C. A. , & Canizales‐Quinteros, S. (2017). An amino acid signature associated with obesity predicts 2‐year risk of hypertriglyceridemia in school‐age children. Scientific Reports, 7(1), 5607–5615. 10.1038/s41598-017-05765-4 28717206 PMC5514079

[fsn31941-bib-0030] Newgard, C. B. , An, J. , Bain, J. R. , Muehlbauer, M. J. , Stevens, R. D. , Lien, L. F. , Haqq, A. M. , Shah, S. H. , Arlotto, M. , Slentz, C. A. , Rochon, J. , Gallup, D. , Ilkayeva, O. , Wenner, B. R. , Yancy, W. S. , Eisenson, H. , Musante, G. , Surwit, R. S. , Millington, D. S. , … Svetkey, L. P. (2009). A branched‐chain amino acid‐related metabolic signature that differentiates obese and lean humans and contributes to insulin resistance. Cell Metabolism, 9(4), 311–325. 10.1016/j.cmet.2009.02.002 19356713 PMC3640280

[fsn31941-bib-0031] Ninio, D. M. , Murphy, K. J. , Howe, P. R. , & Saint, D. A. (2005). Dietary fish oil protects against stretch‐induced vulnerability to atrial fibrillation in a rabbit model. Journal of Cardiovascular Electrophysiology, 16(11), 1189–1194. 10.1111/j.1540-8167.2005.50007.x 16302902

[fsn31941-bib-0032] Parker, H. M. , Cohn, J. S. , O'Connor, H. T. , Garg, M. L. , Caterson, I. D. , George, J. , & Johnson, N. A. (2019). Effect of fish oil supplementation on hepatic and visceral fat in overweight men: A randomized controlled trial. Nutrients, 11(2), 475–490. 10.3390/nu11020475 PMC6413081 30813440

[fsn31941-bib-0033] Parks, D. H. , Tyson, G. W. , Hugenholtz, P. , & Beiko, R. G. (2014). STAMP: Statistical analysis of taxonomic and functional profiles. Bioinformatics, 30(21), 3123–3124. 10.1093/bioinformatics/btu494 25061070 PMC4609014

[fsn31941-bib-0034] Pitcher, M. C. , & Cummings, J. H. (1996). Hydrogen sulphide: A bacterial toxin in ulcerative colitis? Gut, 39, 1–4. 10.1136/gut.39.1.1 8881797 PMC1383219

[fsn31941-bib-0035] Rokling‐Andersen, M. H. , Rustan, A. C. , Wensaas, A. J. , Kaalhus, O. , Wergedahl, H. , Rost, T. H. , & Drevon, C. A. (2009). Marine n‐3 fatty acids promote size reduction of visceral adipose depots, without altering body weight and composition, in male Wistar rats fed a high‐fat diet. British Journal of Nutrition, 102(7), 995–1006. 10.1017/S0007114509353210 19397836

[fsn31941-bib-0036] Sánchez, B. , Delgado, S. , Blanco‐Míguez, A. , Lourenço, A. , Gueimonde, M. , & Margolles, A. (2017). Probiotics, gut microbiota, and their influence on host health and disease. Molecular Nutrition Food Research, 61(1), 1600240 10.1002/mnfr.201600240 27500859

[fsn31941-bib-0037] Saraswathi, V. , Gao, L. , Morrow, J. D. , Chait, A. , Niswender, K. D. , & Hasty, A. H. (2007). Fish oil increases cholesterol storage in white adipose tissue with concomitant decreases in inflammation, hepatic steatosis, and atherosclerosis in mice. The Journal of Nutrition, 137(7), 1776–1782. 10.1093/jn/137.7.1776 17585030

[fsn31941-bib-0038] Sasaki, M. , Ogasawara, N. , Funaki, Y. , Mizuno, M. , Iida, A. , Goto, C. , Koikeda, S. , Kasugai, K. , & Joh, T. (2013). Transglucosidase improves the gut microbiota profile of type 2 diabetes mellitus patients: A randomized double‐blind, placebo‐controlled study. BMC Gastroenterology, 13, 81–88. 10.1186/1471-230X-13-81 23657005 PMC3658914

[fsn31941-bib-0039] Satil, F. , Azcan, N. , & Baser, K. (2003). Fatty acid composition of pistachio nuts in Turkey. Chemistry of Natural Compounds, 39, 322–324. 10.1023/B:CONC.0000003408.63300.b5

[fsn31941-bib-0040] Segata, N. , Izard, J. , Waldron, L. , Gevers, D. , Miropolsky, L. , Garrett, W. S. , & Huttenhower, C. (2011). Metagenomic biomarker discovery and explanation. Genome Biology, 12, R60 10.1186/gb-2011-12-6-r60 21702898 PMC3218848

[fsn31941-bib-0041] Serhan, C. N. (2005). Novel eicosanoid and docosanoid mediators: Resolvins, docosatrienes, and neuroprotectins. Current Opinion in Clinical Nutrition and Metabolic Care, 8(2), 115–121. 10.1097/00075197-200503000-00003 15716788

[fsn31941-bib-0042] Serhan, C. N. (2014). Pro‐resolving lipid mediators are leads for resolution physiology. Nature, 510, 92–101. 10.1038/nature13479 24899309 PMC4263681

[fsn31941-bib-0043] Sharma, S. , & Tripathi, P. (2019). Gut microbiome and type 2 diabetes: Where we are and where to go? The Journal of Nutritional Biochemistry, 63, 101–108. 10.1016/j.jnutbio.2018.10.003 30366260

[fsn31941-bib-0044] Stoner, L. , David, R. , Ariel, M. , Daniel, C. , Michael, H. , Kim, G. , Danielle, L. , & Anna, M. (2016). Efficacy of exercise intervention for weight loss in overweight and obese adolescents: Meta‐analysis and implications. Sports Medicine, 46, 1737–1751. 10.1007/s40279-016-0537-6 27139723

[fsn31941-bib-0045] Sun, W. Z. , Augusto, L. A. , Zhao, L. P. , & Caroff, M. (2015). Desulfovibrio desulfuricans isolates from the gut of a single individual: Structural and biological lipid a characterization. FEBS Letters, 589(1), 165–171. 10.1016/j.febslet.2014.11.042 25479086

[fsn31941-bib-0046] Sundaram, S. , Bukowski, M. R. , Lie, W. R. , Picklo, M. J. , & Yan, L. (2015). High‐fat diets containing different amounts of n3 and n6 polyunsaturated fatty acids modulate inflammatory cytokine production in mice. Lipids, 51(5), 571–582. 10.1007/s11745-015-4093-x 26645280

[fsn31941-bib-0047] Turchini, G. M. , Torstensen, B. E. , & Ng, W. K. (2009). Fish oil replacement in finfish nutrition. Reviews in Aquaculture, 1(1), 10–57. 10.1111/j.1753-5131.2008.01001.x

[fsn31941-bib-0048] Turnbaugh, P. J. , Hamady, M. , Yatsunenko, T. , Cantarel, B. L. , Duncan, A. , Ley, R. E. , Sogin, M. L. , Jones, W. J. , Roe, B. A. , Affourtit, J. P. , Egholm, M. , Henrissat, B. , Heath, A. C. , Knight, R. , & Gordon, J. I. (2009). A core gut microbiome in obese and lean twins. Nature, 457, 480–484. 10.1038/nature07540 19043404 PMC2677729

[fsn31941-bib-0049] Turnbaugh, P. J. , Ley, R. E. , Mahowald, M. A. , Magrini, V. , Mardis, E. R. , & Gordon, J. I. (2006). An obesity‐associated gut microbiome with increased capacity for energy harvest. Nature, 444, 1027–1031. 10.1038/nature05414 17183312

[fsn31941-bib-0050] Van de Velde, L. A. , Subramanian, C. , Smith, A. M. , Barron, L. , Qualls, J. E. , Neale, G. , Alfonso‐Pecchio, A. , Jackowski, S. , Rock, C. O. , Wynn, T. A. , & Murray, P. J. (2017). T cells encountering myeloid cells programmed for amino acid‐dependent immunosuppression use rictor/mTORC2 protein for proliferative checkpoint decisions. The Journal of Biological Chemistry, 292, 15–30. 10.1074/jbc.M116.766238 27903651 PMC5217675

[fsn31941-bib-0051] Wang, Q. X. , Jia, M. Z. , Zhao, Y. H. , Hui, Y. , Pan, J. R. , Yu, H. F. , Yan, S. K. , Dai, X. S. , Liu, X. B. , & Liu, Z. G. (2019). Supplementation of sesamin alleviates stress‐induced behavioral and psychological disorders via reshaping the gut microbiota structure. Journal of Agricultural and Food Chemistry, 67(45), 12441–12451. 10.1021/acs.jafc.9b03652 31674783

[fsn31941-bib-0052] Weglarz, L. , Dzierzewicz, Z. , Skop, B. , Orchel, A. , Parfiniewicz, B. , Wiśniowska, B. , Swiatkowska, L. , & Wilczok, T. (2003). Desulfovibrio desulfuricans lipopolysaccharides induce endothelial cell IL‐6 and IL‐8 secretion and e‐selectin and VCAM‐1 expression. Cellular & Molecular Biology Letter, 8(4), 991–1003. 10.1007/s000180300015 14668922

[fsn31941-bib-0053] Xu, J. , Lian, F. , Zhao, L. , Zhao, Y. , Chen, X. , Zhang, X. , Guo, Y. , Zhang, C. H. , Zhou, Q. , Xue, Z. S. , Pang, X. Y. , Zhao, L. P. , & Tong, X. L. (2015). Structural modulation of gut microbiota during alleviation of type 2 diabetes with a Chinese herbal formula. The ISME Journal, 9, 552–562. 10.1038/ismej.2014.177 25279787 PMC4331591

[fsn31941-bib-0054] Xu, P. F. , Wang, J. L. , Hong, F. , Wang, S. , Jin, X. , Xue, T. T. , Jia, L. , & Zhai, Y. G. (2017). Melatonin prevents obesity through modulation of gut microbiota in mice. Journal of Pineal Research, 62(4), e12399 10.1111/jpi.12399 28199741

[fsn31941-bib-0055] Xu, W. L. , Ghosh, S. , Comhair, S. A. A. , Asosingh, K. , Janocha, A. J. , Mavrakis, D. A. , Bennett, C. D. , Gruca, L. L. , Graham, B. B. , Queisser, K. A. , Kao, C. C. , Wedes, S. H. , Petrich, J. M. , Tuder, R. M. , Kalhan, S. C. , & Erzurum, S. C. (2016). Increased mitochondrial arginine metabolism supports bioenergetics in asthma. The Journal of Clinical Investigation, 126(7), 2465–2481. 10.1172/JCI82925 27214549 PMC4922712

[fsn31941-bib-0056] Yang, J. , & Yu, J. (2018). The association of diet, gut microbiota and colorectal cancer: What we eat may imply what we get? Protein Cell, 9, 474–487. 10.1007/s13238-018-0543-6 29713943 PMC5960467

[fsn31941-bib-0057] Zhang, M. , & Yang, X. J. (2016). Effects of a high fat diet on intestinal microbiota and gastrointestinal diseases. World Journal of Gastroenterology, 22(40), 8905–8909. 10.3748/wjg.v22.i40.8905 27833381 PMC5083795

[fsn31941-bib-0058] Zhu, Z. , Zhu, B. , Sun, Y. , Ai, C. , Wang, L. , Wen, C. , Yang, J. , Song, S. , & Liu, X. (2018). Sulfated polysaccharide from sea cucumber and its depolymerized derivative prevent obesity in association with modification of gut microbiota in high‐fat diet‐fed mice. Molecular Nutrition Food Research, 62, 1800446 10.1002/mnfr.201800446 30267558

